# Analgesic and Hemodynamic Effects of Preoperative Ultrasound-Guided Brachial Plexus Block in Radius Fracture Surgery: A Propensity-Matched Cohort Study

**DOI:** 10.3390/medicina62030493

**Published:** 2026-03-05

**Authors:** Wen-Chen Chao, Han-Yu Lin, Po-Chuan Yu, Ping-Cheng Shih, Meng-Yu Wu, Chun-Yu Chang

**Affiliations:** 1 Department of Anesthesiology, Taipei Tzu Chi Hospital, Buddhist Tzu Chi Medical Foundation, New Taipei City 231, Taiwan; oinfbtc@gmail.com (W.-C.C.); bbkeric@gmail.com (H.-Y.L.); yu136917@gmail.com (P.-C.Y.); benego@gmail.com (P.-C.S.); 2 School of Medicine, Tzu Chi University, Hualien 970, Taiwan; skyshangrila@gmail.com; 3 Department of Emergency Medicine, Taipei Tzu Chi Hospital, Buddhist Tzu Chi Medical Foundation, New Taipei City 231, Taiwan

**Keywords:** brachial plexus block, Kaplan–Meier, opioid-sparing, radius fracture, rebound pain

## Abstract

*Background and Objectives*: Optimal pain control after radius fracture surgery is critical for recovery and reducing opioid exposure. While brachial plexus block (BPB) offers analgesic benefits, its additive effect alongside general anesthesia remains underexplored. *Materials and Methods*: We conducted a retrospective cohort study of adults undergoing open reduction and internal fixation for radius fractures under general anesthesia between July 2020 and September 2025. Patients receiving preoperative ultrasound-guided BPB were matched 1:1 to those without BPB using propensity score matching. Pain scores, hemodynamic changes, and anesthesia-to-incision time were compared using paired *t*-tests or Wilcoxon signed-rank tests. Perioperative opioid consumption and breakthrough morphine use were analyzed using conditional logistic regression, Kaplan–Meier survival analysis, and stratified Cox regression. *Results*: Among 707 eligible patients, 205 who received BPB were matched to 205 controls. In the matched cohort (*n* = 410), BPB was associated with lower intraoperative fentanyl use [16.1% vs. 43.4%; odds ratio (OR) = 0.23; 95% confidence interval (CI): 0.13–0.40; *p* < 0.001], reduced rescue analgesic use in the postanesthesia care unit (10.2% vs. 53.2%; OR = 0.06; 95% CI: 0.03–0.16; *p* < 0.001), and decreased total opioid use (mean morphine milligram equivalents: 2.3 ± 3.4 vs. 6.7 ± 5.6; *p* < 0.001). Pain scores were lower (visual analogue scale: 2.9 ± 1.4 vs. 3.9 ± 1.9; *p* < 0.001). Breakthrough morphine use was delayed and less frequent in the BPB group (19.0% vs. 28.3%; hazard ratio = 0.47; 95% CI: 0.29–0.78; *p* = 0.003). BPB attenuated hemodynamic responses (mean arterial pressure area under curve: 707.2 ± 512.5 vs. 979.3 ± 636.5; *p* < 0.001). *Conclusions*: Preoperative BPB improves perioperative analgesia, lowers opioid use, and stabilizes hemodynamics in radius fracture surgery.

## 1. Introduction

Radius fractures are among the most common fractures of the upper extremity, with distal radius fractures representing the majority of cases [1,2]. Globally, the incidence of radius fractures is rising, particularly in aging populations, contributing substantially to healthcare utilization and disability [3,4]. While most fractures are extra-articular and managed conservatively, surgical intervention becomes more common with intra-articular involvement or increased fracture complexity [1,5,6]. Postoperative pain following radius surgery is a significant clinical concern. Severe acute pain in the early postoperative period has been well-documented and is associated with increased opioid consumption, delayed rehabilitation, prolonged recovery time, and reduced patient satisfaction [7,8]. As a result, optimizing analgesia and perioperative pain management strategies is essential not only for improving clinical outcomes but also for reducing the overall opioid burden [9].

Ultrasound-guided brachial plexus block (BPB) plays a key role in radius fracture surgery by providing regional anesthesia and perioperative analgesia, with higher block success rates and lower complication rates compared to landmark-based techniques [10]. In patients undergoing surgical fixation of radius fractures, BPB alone has been associated with improved early postoperative pain control and reduced opioid requirements compared to general anesthesia alone [11,12]. However, outcomes remain inconsistent. BPB is associated with rebound pain as the blocks wear off, typically within 12 to 24 h postoperatively [12]. One recent study even reported increased opioid use during hospitalization following preoperative single-injection nerve blocks in patients undergoing general anesthesia for arthroplasty and arthroscopic procedures [13].

Most prior studies have compared BPB as a sole anesthetic technique with general anesthesia alone, rather than evaluating BPB as an adjunct to general anesthesia in routine practice [11,12,14,15]. As a result, the incremental benefits of adding BPB, particularly regarding perioperative opioid requirements and hemodynamic modulation, remain incompletely defined. In addition, opioid consumption has commonly been reported as cumulative totals, potentially obscuring clinically meaningful temporal patterns in breakthrough analgesic use. A time-to-event approach enables characterization of when analgesic effects are most pronounced and whether benefits attenuate as block effects resolve. Furthermore, intraoperative hemodynamic effects of adjunctive BPB have rarely been quantified using continuous measures such as area under the curve (AUC). Therefore, we evaluated the analgesic and hemodynamic effects of preoperative ultrasound-guided BPB as an adjunct to general anesthesia in radius fracture surgery, with emphasis on time-dependent opioid use and quantitative hemodynamic assessment.

## 2. Materials and Methods

### 2.1. Study Design

This was a single-center, retrospective cohort study conducted at Taipei Tzu Chi Hospital, New Taipei City, Taiwan. The study protocol was approved by the hospital’s Institutional Review Board (Approval No. 14-IRB126) prior to data collection and analysis. As all data were obtained retrospectively from inpatient medical records and the anesthesia information system without direct patient contact or follow-up, the requirement for written informed consent was waived by the Ethics Committee. To ensure patient confidentiality, all personal identifiers were removed and data were anonymized prior to analysis in accordance with institutional and regulatory guidelines.

### 2.2. Patient Selection

Patients who received surgery for radial fracture under endotracheal general anesthesia between 1 July 2020 and 25 September 2025 were screened for eligibility. Only patients whose clinical data were available and complete prior to the Institutional Review Board approval date were included in the final analysis. Patients were excluded based on the following criteria: age under 18 years; American Society of Anesthesiologists (ASA) physical status classification of 4 or higher; presence of multiple fractures; undergoing more than one surgical procedure during hospitalization; admission to the intensive care unit after surgery; use of patient-controlled analgesia postoperatively; intraoperative infusion of alfentanil; duplicate records; or incomplete clinical data.

### 2.3. Anesthetic Management

All patients underwent general anesthesia with endotracheal intubation. Standard monitoring in accordance with the ASA guidelines was applied prior to induction. Anesthesia was induced using intravenous glycopyrrolate (0.2 mg), dexamethasone (4 mg), fentanyl (1–2 μg/kg), and propofol (1–2 mg/kg). After the loss of consciousness, a neuromuscular blocking agent, either rocuronium (1 mg/kg) or cisatracurium (0.2 mg/kg), was administered to facilitate tracheal intubation, which was performed using a video-assisted laryngoscope.

Anesthesia was maintained with either sevoflurane or desflurane in oxygen/air mixtures, titrated to clinical effect. In the group receiving nerve block, an ultrasound-guided BPB was performed by the attending anesthesiologists or the supervised trainees before the surgical incision using one of three approaches, i.e., interscalene, supraclavicular, or axillary, based on the preference of the anesthesiologists. A total volume of 15–20 mL of 0.25–0.5% ropivacaine was administered. In the non-nerve block group, no regional block was performed. Intraoperative analgesia in both groups was supplemented with intermittent boluses of fentanyl as needed, at the discretion of the attending anesthesiologist. At the conclusion of surgery, neuromuscular blockade was reversed using either neostigmine (0.05 mg/kg) combined with glycopyrrolate (0.01 mg/kg), or sugammadex (2 mg/kg), depending on the muscle relaxant used and clinical considerations.

### 2.4. Postoperative Pain Management

In the postanesthesia care unit (PACU), patients who reported pain received rescue analgesics as needed, including intravenous morphine, fentanyl, tramadol, or ketorolac, at the discretion of the attending anesthesiologist. Following transfer to the ward, all patients received oral acetaminophen 500 mg four times daily as a baseline analgesic. Additional analgesia with intramuscular morphine was administered on an as-needed basis for breakthrough pain.

### 2.5. Data Collection

Data were collected from electronic medical records and anesthesia charts. Patient-related variables included age, gender, and ASA physical status classification. Comorbidities were recorded and used to calculate the Charlson Comorbidity Index (CCI), including the presence of myocardial infarction, congestive heart failure, peripheral vascular disease, cerebrovascular disease, dementia, chronic pulmonary disease, rheumatologic disease, peptic ulcer disease, liver disease, diabetes mellitus, hemiplegia or paraplegia, renal disease, malignancy, leukemia, lymphoma, and acquired immunodeficiency syndrome [16]. Intraoperative variables included total anesthesia duration, operative time, interval from anesthesia induction to surgical incision, estimated blood loss, emergency surgery status, fracture site, BPB approach, volume and concentration of ropivacaine administered for the block, use of vasoactive agents, and administration of preemptive analgesics. Hemodynamic data, including heart rate, systolic blood pressure (SBP), diastolic blood pressure (DBP), and mean arterial pressure (MAP), were extracted at fixed 5 min intervals, beginning at the time point closest to 5 min prior to surgical incision and continuing for a total duration of one hour. Postoperative variables included the use and dosage of rescue analgesics administered in the PACU, the first recorded visual analogue scale (VAS) pain score upon arrival at the ward, use and cumulative dose of breakthrough intramuscular morphine in the ward, time from anesthesia induction to the first dose of morphine in the ward, and total length of hospital stay. Records with incomplete data for key study variables were excluded from the final analysis.

### 2.6. Statistical Analysis

Continuous variables were assessed for normality using skewness and kurtosis. An absolute skewness > 2 or absolute kurtosis > 7 was considered indicative of non-normality [17,18]. Normally distributed continuous variables were reported as mean ± standard deviation, while non-normally distributed variables were presented as median (interquartile range [IQR]). Categorical variables were summarized as counts and percentages. For between-group comparisons, two-sided *t*-tests were used for normally distributed continuous variables, and Wilcoxon rank-sum tests for non-normally distributed ones. Chi-square tests were applied to categorical data, with Fisher’s exact test used when expected cell counts were less than 5. Covariate balance between groups was assessed using standardized mean differences (SMDs), with values < 0.1 considered indicative of acceptable balance [19]. Propensity scores were estimated using logistic regression models that included gender, ASA physical status, CCI, emergency surgery status, use of preemptive parecoxib and paracetamol, fracture site, operative time, estimated blood loss, and year or the operation. Propensity score matching (PSM) was performed using the genetic matching algorithm with a 1:1 ratio [20]. Covariate balance after matching was assessed using density plots and SMDs.

Opioid consumption was quantified using intravenous morphine milligram equivalents (MME). Conversion factors of 0.1 were applied for both tramadol and fentanyl based on published equianalgesic data [21,22,23]. Although equianalgesic ratios vary across sources and are influenced by interindividual pharmacokinetic variability, standardization to morphine equivalents is widely accepted in perioperative opioid consumption analyses. Total MME was defined as the cumulative dose of all opioid analgesics administered after anesthesia induction until hospital discharge, excluding fentanyl used for intubation during induction. Because some patients did not receive any intraoperative or postoperative opioids, total MME data were also summarized among patients with nonzero values. The time to the first dose of breakthrough morphine in the ward was summarized only among patients who received breakthrough morphine, and categorized into five intervals: <6 h, 6–12 h, 12–18 h, 18–24 h, and >24 h. For comparisons after PSM, paired *t*-tests or Wilcoxon signed-rank tests were used for continuous variables, while McNemar’s or Bowker’s test was used for categorical variables. Conditional logistic regression was used to evaluate intraoperative fentanyl administration, use of intraoperative vasoactive agents, intraoperative hypertension and hypotension, rescue analgesic use in the PACU, and any opioid use from anesthesia induction until hospital discharge. Variables which remained imbalanced (with SMD ≥ 0.1) were included in the multivariable regression analyses. Intraoperative hypertension and hypotension were defined as a SBP or MAP ≥ 20% above or ≤20% below the value recorded closest to 5 min prior to surgical incision, respectively. Hemodynamic changes were also analyzed by plotting group-level trends over time. Baseline was defined as the time point closest to 5 min prior to surgical incision. The AUC for deviations from baseline was calculated using the trapezoidal integration method.

Kaplan–Meier curves were plotted to assess time to first use of breakthrough morphine in the ward and were compared using the log-rank test. Median follow-up time in both groups were estimated using the reverse Kaplan–Meier method [24]. Stratified Cox proportional hazards regression was used to estimate the effect of nerve block on time to breakthrough morphine, accounting for matched pairs. The proportional hazards assumption was evaluated using the Grambsch–Therneau global test for non-proportionality [25]. When the assumption was violated, time-varying effects were modelled using two approaches: an interaction between the exposure variable and log-transformed time (log-time model), and a flexible penalized spline of log-time (spline model) to allow for non-linear hazard ratio trajectories over time [26,27]. Model fit was compared using the Akaike Information Criterion (AIC).

A subgroup analysis was conducted to evaluate the association between BPB approach, ropivacaine dose, and ropivacaine volume with the outcomes of interest using multivariable regression models. To minimize collinearity, this analysis was restricted to patients who received ultrasound-guided BPB. A sensitivity analysis was further performed to assess the potential impact of BPB failure on the primary outcomes. Assumed block failure rates of 5%, 10%, and 15% were evaluated using Monte Carlo simulation with 500 iterations per scenario. For each assumed failure rate, a corresponding proportion of patients in the BPB group was randomly reclassified as non-BPB. Propensity score matching was then repeated, followed by outcome analyses using the same regression models as in the primary analysis. For each failure-rate scenario, results were summarized as the median effect estimate, empirical 95% interval (2.5th–97.5th percentiles), and the proportion of simulations yielding statistical significance. A *p*-value < 0.05 was considered statistically significant throughout the study. All statistical analyses were performed using R version 4.4.2, primarily utilizing the “MatchIt (version 4.7.2)”, “survival (version 3.8-6)” and “ggplot2 (version 4.0.2)” packages [28].

## 3. Results

### 3.1. Patient Selection Flow Diagram

**Figure 1:**
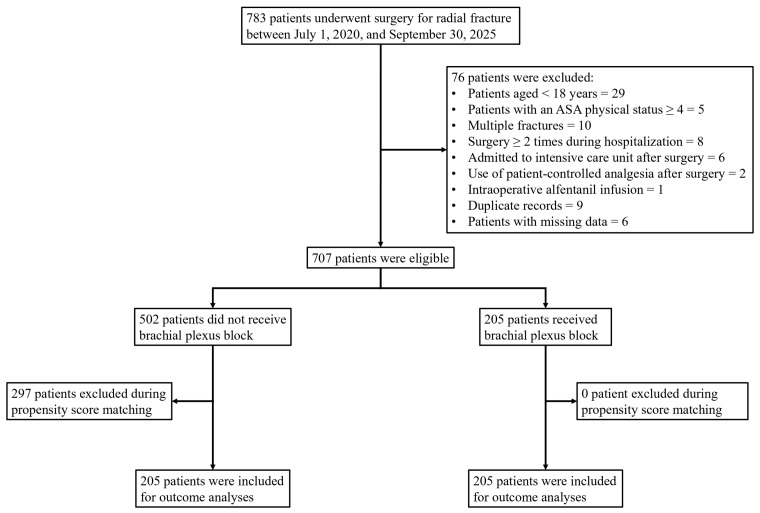
Patient selection flow diagram.

The patient selection process is illustrated in Figure 1. A total of 783 patients who underwent surgery for radial fracture between 1 July 2020 and 30 September 2025 were identified. Seventy-six patients were excluded for the following reasons: age under 18 years (*n* = 29); ASA physical status classification ≥ 4 (*n* = 5); presence of multiple fractures (*n* = 10); undergoing more than one surgical procedure during hospitalization (*n* = 8); postoperative admission to the intensive care unit (*n* = 6); use of patient-controlled analgesia (*n* = 2); intraoperative infusion of alfentanil (*n* = 1); duplicate records (*n* = 9); and incomplete clinical data (*n* = 6). After exclusions, 707 patients were eligible for analysis, of whom 205 received an ultrasound-guided BPB prior to surgical incision, and 502 did not. Following PSM at a 1:1 ratio, all 205 patients who received BPB were successfully matched to 205 patients without BPB, leaving 297 unmatched patients excluded. A total of 410 patients (205 per group) were included in the final outcome analyses.

### 3.2. Patient Demographics

Before PSM, patients who received BPB were more likely to receive preemptive analgesics (64.9% vs. 46.6%), including parecoxib and paracetamol, and were more frequently treated in emergent surgical settings. These imbalances were reflected in the SMDs, particularly for preemptive analgesia (SMD = 0.374), parecoxib use (SMD = 0.317), and paracetamol use (SMD = 0.396). After 1:1 PSM, 205 patients remained in each group. Baseline characteristics between the matched groups were mostly balanced with SMDs below 0.1. This improvement in balance was also confirmed visually using density plots of the propensity score distribution, which showed substantial overlap between groups after matching (Supplementary Figure S1). The mean age was approximately 61 years in both groups, with similar distributions in gender, ASA physical status, CCI, and use of preemptive analgesics (Table 1).

### 3.3. Intraoperative and Postoperative Outcomes

Intraoperative fentanyl use was significantly lower in the BPB group compared with the non-BPB group (16.1% vs. 43.4%; *p* < 0.001). In univariable analysis, BPB was associated with reduced odds of fentanyl administration (cOR [crude odds ratio] 0.27, 95% CI [confidence interval] 0.17–0.44; *p* < 0.001), which remained significant after adjustment (aOR [adjusted odds ratio] 0.23, 95% CI 0.13–0.40; *p* < 0.001). Rescue analgesics use in the PACU was markedly lower in patients receiving BPB (10.2% vs. 53.2%; cOR 0.08, 95% CI 0.04–0.17; *p* < 0.001), with consistent findings in multivariable analysis (aOR 0.06, 95% CI 0.03–0.16; *p* < 0.001). Opioid use during hospitalization was similarly reduced in the BPB group (37.1% vs. 78.5%; cOR 0.16, 95% CI 0.09–0.27; *p* < 0.001; aOR 0.10, 95% CI 0.05–0.21; *p* < 0.001). Regarding intraoperative hemodynamics, BPB was associated with a lower incidence of hypertension (57.6% vs. 77.1%; cOR 0.39, 95% CI 0.24–0.61; *p* < 0.001; aOR 0.38, 95% CI 0.23–0.63; *p* < 0.001). In contrast, hypotension rates did not differ significantly between groups (21.5% vs. 17.1%; aOR 1.46, 95% CI 0.80–2.68; *p* = 0.221). BPB was also associated with lower odds of intraoperative labetalol use (aOR 0.11, 95% CI 0.02–0.50; *p* = 0.005) and nicardipine use (aOR 0.46, 95% CI 0.23–0.91; *p* = 0.025), whereas ephedrine use was more frequent in the BPB group (aOR 7.45, 95% CI 2.27–24.4; *p* < 0.001). The mean MME administered in the PACU was reduced (0.5 ± 1.6 vs. 2.8 ± 3.2; *p* < 0.001), as was total MME during hospitalization (2.3 ± 3.4 vs. 6.7 ± 5.6; *p* < 0.001). Among patients who required any opioids, total MME remained significantly lower in the BPB group (6.2 ± 2.8 vs. 8.6 ± 5.0; *p* < 0.001). The first recorded VAS pain score in the ward was also lower in the BPB group (2.9 ± 1.4 vs. 3.9 ± 1.9; *p* < 0.001). Breakthrough morphine use in the ward occurred less frequently in the BPB group (19.0% vs. 28.3%; *p* = 0.026). Among patients requiring breakthrough morphine, those in the BPB group tended to receive it later. In the non-BPB group, 65.5% required morphine within 6 h of anesthesia induction compared with 17.9% in the BPB group, whereas 35.9% of BPB patients received breakthrough morphine between 12 and 18 h post-induction compared with 3.5% of non-BPB patients. Additionally, the interval between anesthesia induction and surgical incision was slightly longer in the BPB group (44.3 ± 9.3 vs. 38.0 ± 9.3 min, *p* < 0.001). All intraoperative and postoperative outcomes are summarized in Table 2, and detailed conditional logistic regression analyses are presented in Supplementary Table S1.

### 3.4. Time-to-Event Analysis for Breakthrough Morphine Use in the Ward

**Figure 2:**
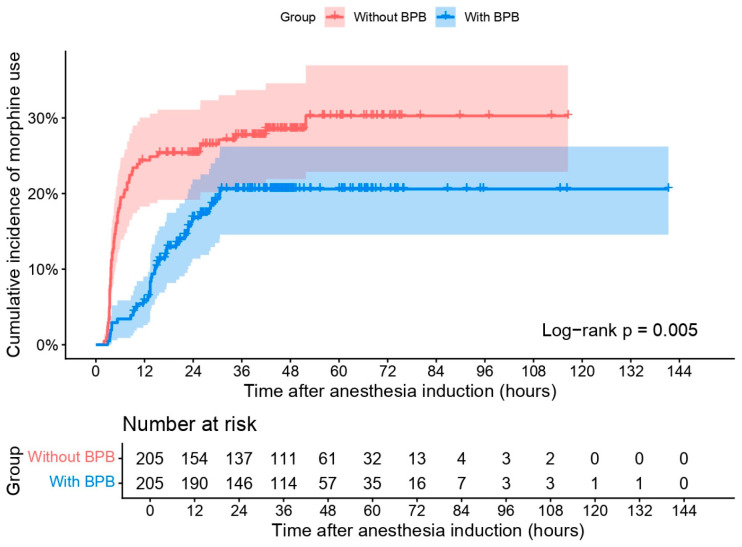
Kaplan–Meier plot of the cumulative incidence of breakthrough morphine use in the ward. BPB: brachial plexus block.

The median follow-up time for breakthrough morphine use in the ward was 44.7 h (IQR 35.9–56.3) in the non-BPB group and 44.0 h (IQR 29.5–50.2) in the BPB group. Patients who received BPB demonstrated a significantly lower and delayed cumulative incidence of breakthrough morphine use compared with those without BPB (Figure 2). Within the first 12 h after anesthesia induction, the non-BPB group showed a rapid increase in morphine use, reaching approximately 30%, whereas the BPB group remained below 15% during the same period. The cumulative incidence plateaued earlier and at a lower level in the BPB group (~20%) than in the non-BPB group (~30%). The difference between groups was statistically significant (log-rank *p* = 0.005).

**Figure 3:**
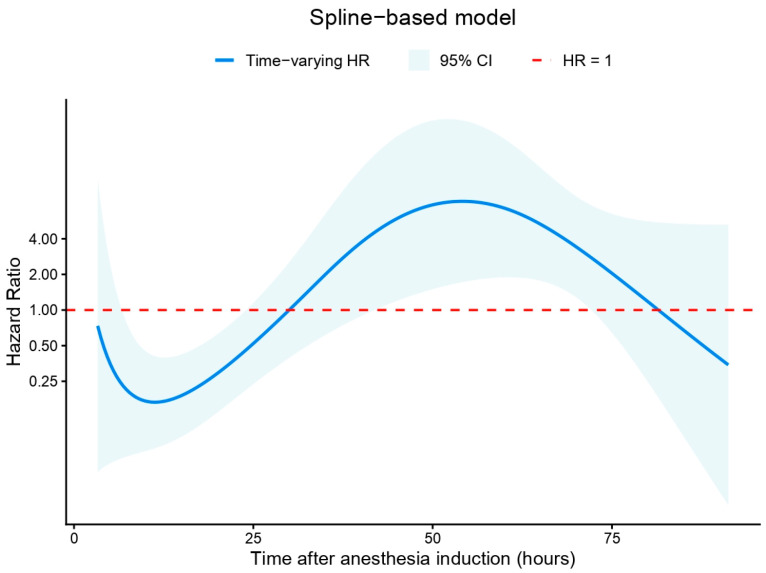
Time-varying hazard ratio with a spline-based model. The spline-based model demonstrated a non-linear time-varying hazard ratio. The hazard ratio was initially well below 1, increased over time, and approached or exceeded 1 between approximately 40 and 60 h. Confidence bands widened in later time intervals. CI: confidence interval. HR: hazard ratio.

In the univariable stratified Cox regression model, BPB was associated with a significantly reduced hazard of requiring breakthrough morphine in the ward (cHR [crude hazard ratio] 0.53; 95% CI 0.34–0.83; *p* = 0.005) (Supplementary Table S2). After adjustment, the association remained significant (aHR 0.47; 95% CI 0.29–0.78; *p* = 0.003). However, the proportional hazards assumption was violated (Grambsch–Therneau test, *p* < 0.001), indicating a time-dependent effect. To address this, two time-varying models were constructed. In the log-time interaction model (Supplementary Figure S2), the interaction term was statistically significant (HR 4.61; 95% CI 2.21–9.62; *p* < 0.001), and the upper bound of the 95% CI crossed the null at approximately 10.5 h after anesthesia induction. The spline-based model (Figure 3) demonstrated a non-linear time-varying hazard ratio. The HR was initially well below 1, increased over time, and approached or exceeded 1 between approximately 40 and 60 h. Confidence bands widened in later time intervals. Model comparison based on AIC favored the spline-based model (AIC 87.6) over the log-time model (AIC 91.1).

### 3.5. Area Under Curve of Hemodynamic Changes

**Figure 4:**
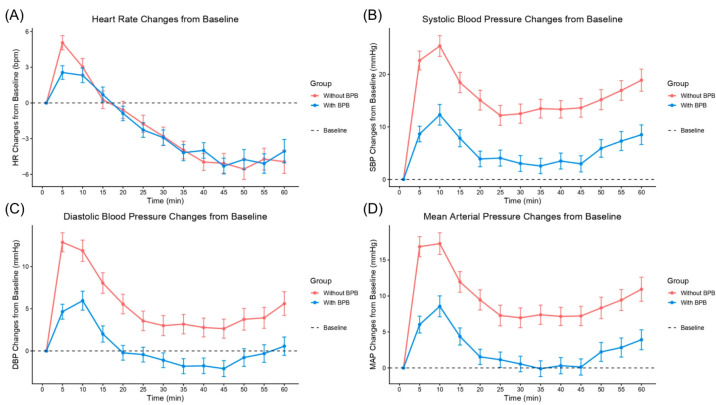
Hemodynamic changes from baseline over time. (**A**) Heart rate changes from baseline. (**B**) Systolic blood pressure changes from baseline. (**C**) Diastolic blood pressure changes from baseline. (**D**) Mean arterial pressure changes from baseline. BPB: brachial plexus block. DBP: diastolic blood pressure. HR: heart rate. MAP: mean arterial pressure. SBP: systolic blood pressure.

Changes in heart rate, SBP, DBP, and MAP from baseline were measured at 5 min intervals from immediately before surgical incision up to 60 min (Figure 4). The mean AUC for heart rate was significantly lower in the BPB group compared with the non-BPB group (419.3 ± 320.8 vs. 504.5 ± 299.4; *p* = 0.006). Similarly, the AUC for SBP was reduced in the BPB group (972.7 ± 707.4 vs. 1360.1 ± 890.8; *p* < 0.001). For DBP, the AUC was 582.2 ± 387.1 in the BPB group compared with 789.3 ± 496.2 in the non-BPB group (*p* < 0.001). The AUC for MAP was likewise significantly lower in the BPB group (707.2 ± 512.5 vs. 979.3 ± 636.5; *p* < 0.001) (Supplementary Table S3).

### 3.6. Subgroup Analysis

A subgroup analysis restricted to patients receiving ultrasound-guided BPB was performed to explore potential heterogeneity by block approach, ropivacaine dose, and injectate volume (Supplementary Tables S4–S6). Overall, most associations between BPB approach and clinical outcomes were not statistically significant and were characterized by wide confidence intervals, reflecting limited sample size within each approach subgroup. Higher ropivacaine dose (per 10 mg) was associated with lower odds of intraoperative fentanyl use (aOR 0.70, 95% CI 0.49–0.98; *p* = 0.041) and reduced opioid use during hospitalization (aOR 0.76, 95% CI 0.58–0.98; *p* = 0.038). However, dose was not consistently associated with other outcomes. Injectate volume and block approach demonstrated variable associations across outcomes, with several estimates showing wide confidence intervals and marked imprecision, suggesting potential instability due to sparse events. These findings should therefore be interpreted cautiously as exploratory analyses rather than definitive evidence of differential effects among BPB techniques.

### 3.7. Sensitivity Analyses

To evaluate the potential impact of BPB failure misclassification, Monte Carlo simulations were conducted assuming failure rates of 5%, 10%, and 15%, with 500 iterations per scenario (Supplementary Table S7). For binary outcomes, the median effect estimates remained directionally consistent with the primary analysis across all simulated failure rates. Intraoperative fentanyl use, rescue analgesic use in the PACU, and opioid use during hospitalization remained significantly reduced in the BPB group in 100% of simulations across all failure scenarios. Similarly, intraoperative hypertension and labetalol use demonstrated stable effect estimates with high significance retention (>97%). For breakthrough morphine use in the ward, the hazard ratio remained below 1 across simulated failure rates (median HR 0.57–0.77), although the proportion of statistically significant simulations decreased with increasing failure assumptions (80.0% at 5%, 72.4% at 10%, and 67.8% at 15%). Continuous outcomes, including MME at PACU, total MME, and first VAS score in the ward, demonstrated stable median differences with 100% significance retention across all simulated scenarios. Overall, these findings indicate that the primary results were robust to plausible BPB failure rates up to 15%.

## 4. Discussion

This propensity score-matched cohort study demonstrated that preoperative ultrasound-guided BPB, when added to general anesthesia for radius fracture surgery, was associated with substantial improvements in perioperative analgesic and hemodynamic outcomes. Compared with general anesthesia alone, BPB significantly reduced intraoperative fentanyl use (aOR 0.23), rescue analgesic use in the PACU (aOR 0.06), and overall opioid exposure during hospitalization (aOR 0.10). Total opioid consumption was reduced by approximately 4–5 MME, and early postoperative pain scores were lower by an average of 1 point on the VAS. Breakthrough morphine use in the ward was both less frequent (aHR 0.47) and temporally delayed, with time-varying analyses indicating that the protective association was strongest within the first 10–12 h after anesthesia induction. At later time points, effect estimates became less precise, with widening confidence intervals reflecting fewer remaining at-risk events rather than clear evidence of reversal of treatment effect. In addition, BPB attenuated intraoperative hemodynamic responses, reflected by lower incidence of hypertension and reduced area under the curve for heart rate and blood pressure deviations. Although BPB modestly prolonged the induction-to-incision interval by approximately 6 min, this delay was small in magnitude. Sensitivity analyses accounting for plausible block failure rates up to 15% confirmed the robustness of the primary findings.

Our findings align with previous studies indicating that combining regional anesthesia with general anesthesia provides superior analgesia and hemodynamic stability compared with general anesthesia alone [29,30,31]. Surgical stimulation activates peripheral nociceptors, generating afferent signals that ascend through the spinal cord to higher centers where they are perceived as pain and concurrently trigger sympathetic activation, leading to increases in heart rate and blood pressure [32,33]. By reversibly blocking voltage-gated sodium channels, local anesthetics interrupt action potential propagation at the peripheral nerve level, thereby attenuating nociceptive transmission and the associated autonomic responses [34]. These physiological mechanisms were reflected in our findings. Patients who received BPB had a markedly lower incidence of intraoperative fentanyl use, reduced need for PACU rescue analgesia, and attenuated hemodynamic responses during the surgery. In addition, BPB was associated with a reduction in total opioid consumption of approximately 4–5 MME and an improvement in early postoperative pain scores of 1.0 points on the VAS. While the observed opioid reduction did not reach commonly cited minimal clinically important difference (MCID) thresholds (e.g., ≥10 MME or 40% relative reduction), the decrease in VAS score approached the lower bound of reported MCID values (1.0–1.8 cm or approximately 30% relative change) [35,36]. Although the absolute opioid reduction was modest, the prior literature suggests that even small decreases in intravenous morphine exposure may be associated with fewer opioid-related adverse effects [37]. Therefore, these findings should be interpreted as statistically significant and potentially clinically meaningful opioid-sparing effects, albeit of moderate magnitude. Collectively, BPB appears to provide improved analgesic control and hemodynamic stability when used adjunctively with general anesthesia.

The delayed and reduced incidence of breakthrough morphine use in the BPB group suggests that analgesic benefit extends beyond the intraoperative period, with the strongest reduction in hazard observed during the first 10–12 h after anesthesia induction. Beyond this window, effect estimates became less precise as the number of patients remaining at risk declined, partly reflecting increasing statistical uncertainty rather than clear reversal of treatment benefit. This temporal pattern is also clinically consistent with the phenomenon of rebound pain, commonly defined as acute postoperative pain that emerges following resolution of a peripheral nerve block and reaches clinically significant intensity at the surgical site once regional anesthesia dissipates [38,39,40]. Rebound pain typically develops within 12–24 h after block placement, frequently during the first postoperative night, and may persist for several hours before stabilizing [38,41]. Orthopedic procedures involving bone surgery represent a particularly high-risk setting, especially in younger patients and those undergoing single-shot rather than continuous techniques [40,41,42]. Mechanistically, rebound pain is not necessarily attributable to the block itself but may reflect the abrupt unmasking of the underlying nociceptive trajectory once dense afferent inhibition resolves. Proposed mechanisms in the literature include insufficient overlap of systemic multimodal analgesia, transient nociceptive hypersensitivity, peripheral and central sensitization, inflammatory mediator upregulation, and heightened C-fiber excitability following surgical trauma [38,40,43]. Importantly, this does not imply that regional anesthesia worsens overall pain outcomes; rather, it underscores the time-limited pharmacodynamic profile of single-injection blocks. The clinical implication aligns with current recommendations for rebound pain mitigation, including scheduled multimodal regimens, timely oral analgesic administration, patient education regarding anticipated sensory return, and consideration of adjuncts such as intravenous dexamethasone or continuous catheter techniques in higher-risk cases [40].

Ultrasound-guided BPB is a precise procedure that requires adequate time to ensure efficacy and minimize complications. Consequently, concerns have been raised that its use may delay surgical start times and reduce operating room efficiency. In a U.S. cohort undergoing elective surgery, the median time from anesthesia start to procedure initiation was reported as 38 min [44]. In patients receiving peripheral nerve blocks for total knee arthroplasty, the induction-to-incision interval was 55 min compared to 47 min in those without a block [45]. In our study, the average time from anesthesia induction to surgical incision was 44.3 min in the BPB group and 38.0 min in the non-BPB group, a statistically significant difference of 5.3 min. However, this modest delay is consistent with previously published timeframes and remains well below any validated threshold for clinical impact.

Despite the favorable findings, several limitations should be acknowledged. First, although propensity score matching improved group comparability, this retrospective observational design remains vulnerable to residual confounding. Important perioperative factors such as anesthesiologist experience, block performance quality, intraoperative anesthetic depth, and adjunct analgesic strategies were not fully captured and may have influenced outcomes; therefore, causal inference should be made cautiously. Second, the BPB intervention was heterogeneous with respect to block approach and local anesthetic dose and volume, which may affect block duration, hemodynamic responses, and rebound pain. Although subgroup analyses were conducted, sparse events and wide confidence intervals limited definitive interpretation. In addition, formal assessment of block success was not uniformly documented, raising the possibility of exposure misclassification despite sensitivity analyses addressing plausible block failure rates. Third, the time-to-event endpoint reflected clinician-administered breakthrough morphine rather than pain intensity alone, introducing potential treatment-decision bias. While later hazard ratio estimates became less precise, this reflects increasing statistical uncertainty over time rather than clear reversal of effect. Fourth, the six-year inclusion period may have encompassed evolving perioperative practices. Year of surgery was incorporated into the propensity model and balanced after matching; however, unmeasured temporal changes cannot be excluded. Similarly, although fracture site, operative time, and blood loss were adjusted for, detailed surgical complexity and intraoperative variability were not fully characterized. Finally, this single-center study was conducted within a specific regional anesthesia workflow, which may limit generalizability to institutions with different expertise or perioperative protocols. Outcomes were confined to the in-hospital period; long-term pain, functional recovery, and quality-of-recovery metrics were not assessed.

## 5. Conclusions

In conclusion, preoperative ultrasound-guided BPB administered in conjunction with general anesthesia for radius fracture surgery was associated with improved perioperative analgesia, reduced opioid exposure, and attenuated intraoperative hemodynamic responses. The analgesic benefit was most pronounced within the first 10–12 h after anesthesia induction, consistent with the expected pharmacodynamic profile of single-injection regional anesthesia. Although the induction-to-incision interval was modestly longer in patients receiving BPB, the absolute difference was small and should be interpreted in the context of overall perioperative workflow rather than against a validated threshold for clinical relevance. Taken together, BPB may serve as a useful adjunct to general anesthesia in radius fracture surgery, particularly for enhancing early postoperative analgesia and reducing opioid requirements. The time-dependent attenuation of benefit underscores the importance of structured transitional multimodal analgesia as block effects resolve. Prospective, multicenter studies are warranted to confirm these findings, clarify optimal block strategies, and evaluate longer-term patient-centered outcomes.

## Figures and Tables

**Table 1:** Patient demographics.

	Overall Cohort (*n* = 707)	Unmatched Cohort				Matched Cohort		
		Without BPB (*n* = 502)	With BPB (*n* = 205)	*p* Value	SMD	Without BPB (*n* = 205)	With BPB (*n* = 205)	SMD
Age	62.2 ± 15.2	62.8 ± 14.4	60.7 ± 17.0	0.125	0.132	61.4 ± 14.5	60.7 ± 17.0	0.047
Gender								
Female	520 (73.6)	370 (73.7)	150 (73.2)	0.884	0.012	146 (71.2)	150 (73.2)	0.044
Male	187 (26.4)	132 (26.3)	55 (26.8)			59 (28.8)	55 (26.8)	
Year								
2020–2022	310 (43.8)	270 (53.8)	40 (19.5)	<0.001	0.761	45 (22.0)	40 (19.5)	0.060
2023–2025	397 (56.2)	232 (46.2)	165 (80.5)			160 (78.0)	165 (80.5)	
Emergent surgery	456 (64.5)	311 (62.0)	145 (70.7)	0.027	0.187	147 (71.7)	145 (70.7)	0.022
Fracture site								
Non-distal	71 (10.0)	49 (9.8)	22 (10.7)	0.697	0.032	16 (7.8)	22 (10.7)	0.101
Distal	636 (90.0)	453 (90.2)	183 (89.3)			189 (92.2)	183 (89.3)	
Preemptive analgesics	367 (51.9)	234 (46.6)	133 (64.9)	<0.001	0.374	130 (63.4)	133 (64.9)	0.031
Parecoxib	318 (45.0)	203 (40.4)	115 (56.1)	<0.001	0.317	115 (56.1)	115 (56.1)	0.000
Paracetamol	337 (47.7)	211 (42.0)	126 (61.5)	<0.001	0.396	122 (59.5)	126 (61.5)	0.040
CCI	2.5 ± 1.6	2.6 ± 1.6	2.4 ± 1.7	0.213	0.104	2.4 ± 1.5	2.4 ± 1.7	0.018
ASA PS								
1	25 (3.5)	17 (3.4)	8 (3.9)	0.944	0.028	8 (3.9)	8 (3.9)	0.060
2	589 (83.3)	419 (83.5)	170 (82.9)			174 (84.9)	170 (82.9)	
3	93 (13.2)	66 (13.1)	27 (13.2)			23 (11.2)	27 (13.2)	
Anesthesia time	140.0 (120.0–165.0)	140.0 (117.0–168.5)	140.0 (122.0–161.0)	0.916	0.043	135.0 (110.0–155.0)	140.0 (122.0–161.0)	0.242
Operative time	90.0 (70.0–110.0)	90.0 (70.0–115.0)	85.0 (70.0–107.0)	0.086	0.153	85.0 (68.0–105.0)	85.0 (70.0–107.0)	0.073
Blood loss	10.0 (5.0–10.0)	10.0 (5.0–10.0)	10.0 (5.0–10.0)	0.164	0.088	10.0 (5.0–10.0)	10.0 (5.0–10.0)	0.035
BPB approach								
Interscalene	25 (3.5)		25 (12.2)	<0.001	1.049		25 (12.2)	1.049
Supraclavicular	131 (18.5)		131 (63.9)				131 (63.9)	
Axillary	49 (6.9)		49 (23.9)				49 (23.9)	

Data are presented as mean ± standard deviation, median (interquartile range), or count (frequency). ASA PS: American Society of Anesthesiologists physical status; BPB: brachial plexus block; CCI: Charlson comorbidity index; SMD: standardized mean difference.

**Table 2:** Intraoperative and postoperative outcomes ^1^.

	Without BPB (*n* = 205)	With BPB (*n* = 205)	*p* Value
Intraoperative fentanyl use	89 (43.4)	33 (16.1)	<0.001
Intraoperative ephedrine use	8 (3.9)	33 (16.1)	<0.001
Intraoperative nicardipine use	29 (14.1)	15 (7.3)	0.027
Intraoperative labetalol use	27 (13.2)	7 (3.4)	<0.001
Intraoperative hypertension	158 (77.1)	118 (57.6)	<0.001
Intraoperative hypotension	35 (17.1)	44 (21.5)	0.225
Rescue analgesics use at PACU ^2^	109 (53.2)	21 (10.2)	<0.001
Opioid analgesics use during hospitalization	161 (78.5)	76 (37.1)	<0.001
Breakthrough morphine use in the ward	58 (28.3)	39 (19.0)	0.026
Time to first requirement of breakthrough morphine in the ward (nonzero)			
<6 h	38 (65.5)	7 (17.9)	<0.001
6–12 h	12 (20.7)	5 (12.9)	
12–18 h	2 (3.5)	14 (35.9)	
18–24 h	0 (0.0)	7 (17.9)	
>24 h	6 (10.3)	6 (15.4)	
MME at PACU ^3^	2.8 ± 3.2 2.0 (0.0–5.0)	0.5 ± 1.6 0.0 (0.0–0.0)	<0.001 <0.001
Total MME ^3^	6.7 ± 5.6 5.0 (2.5–10.0)	2.3 ± 3.4 0.0 (0.0–5.0)	<0.001 <0.001
Total MME (nonzero) ^3^	8.6 ± 5.0 7.5 (5.0–10.0)	6.2 ± 2.8 5.0 (5.0–9.3)	<0.001 <0.001
First VAS pain score in the ward	3.9 ± 1.9	2.9 ± 1.4	<0.001
Interval between anesthesia induction and surgical incision	38.0 ± 9.3	44.3 ± 9.3	<0.001
Hospitalization day	3.0 (2.0–3.0)	2.0 (2.0–3.0)	0.308

^1^ Data are presented as mean ± standard deviation, median (interquartile range), or count (frequency). ^2^ Rescue analgesics at PACU included intravenous morphine, fentanyl, tramadol, or ketorolac. ^3^ Both mean ± standard deviation and median (interquartile range) are presented. BPB: brachial plexus block; MME: morphine milligram equivalents; PACU: postanesthesia care unit; VAS: visual analogue scale.

## Data Availability

The data presented in this study are available on reasonable request from the corresponding author. The data are not publicly available due to privacy and ethical restrictions.

## Supplementary Materials

The following supporting information can be downloaded at https://www.mdpi.com/article/10.3390/medicina62030493/s1, Supplementary Figure S1: (A) Propensity score distribution in both groups before matching. (B) Propensity score distribution in both groups after matching. BPB: brachial plexus block; Supplementary Figure S2: Time-varying hazard ratio with a log-time interaction model. CI: confidence interval. HR: hazard ratio; Supplementary Table S1: Univariable conditional logistic regression; Supplementary Table S2: Univariable stratified cox regression; Supplementary Table S3: Area under curve of hemodynamic changes. Supplementary Table S4: Association of brachial plexus block approaches, ropivacaine dose and volume on binary outcomes. Supplementary Table S5: Association of brachial plexus block approaches, ropivacaine dose and volume on continuous outcomes. Supplementary Table S6: Association of brachial plexus block approaches, ropivacaine dose and volume on time-to-event outcomes. Supplementary Table S7: Sensitivity analysis assessing the potential impact of brachial plexus block failure on the primary outcomes.

## Abbreviations

The following abbreviations are used in this manuscript:

AIC	Akaike information criterion
ASA	American Society of Anesthesiologists
AUC	Area under curve
BPB	Brachial plexus block
CCI	Charlson comorbidity index
CI	Confidence interval
DBP	Diastolic blood pressure
HR	Hazard ratio
IQR	Interquartile range
MAP	Mean arterial pressure
MCID	Minimal clinically important difference
MME	Morphine milligram equivalents
NMA	Nociceptive medullary autonomic
OR	Odds ratio
PACU	Postanesthesia care unit
PSM	Propensity score matching
SBP	Systolic blood pressure
SMD	Standardized mean difference
VAS	Visual analogue scale
