# Supplementary material for: Analgesic and Hemodynamic Effects of Preoperative Ultrasound-Guided Brachial Plexus Block in Radius Fracture Surgery: A Propensity-Matched Cohort Study
Source: Medicina (Kaunas). 2026 Mar 5;62(3):493. doi: 10.3390/medicina62030493 (PMC13028216; doi:10.3390/medicina62030493)
Supplement: Supplementary file 1 [file medicina-62-00493-s001.zip › medicina-4138953-supplementary.pdf]

Supplementary Materials

Analgesic and Hemodynamic Effects of Preoperative Ultrasound-Guided Brachial Plexus Block in Radius Fracture Surgery: A Propensity-Matched Cohort Study

Wen-Chen Chao, Han-Yu Lin, Po-Chuan Yu, Ping-Cheng Shih, Meng-Yu Wu, Chun-Yu Chang

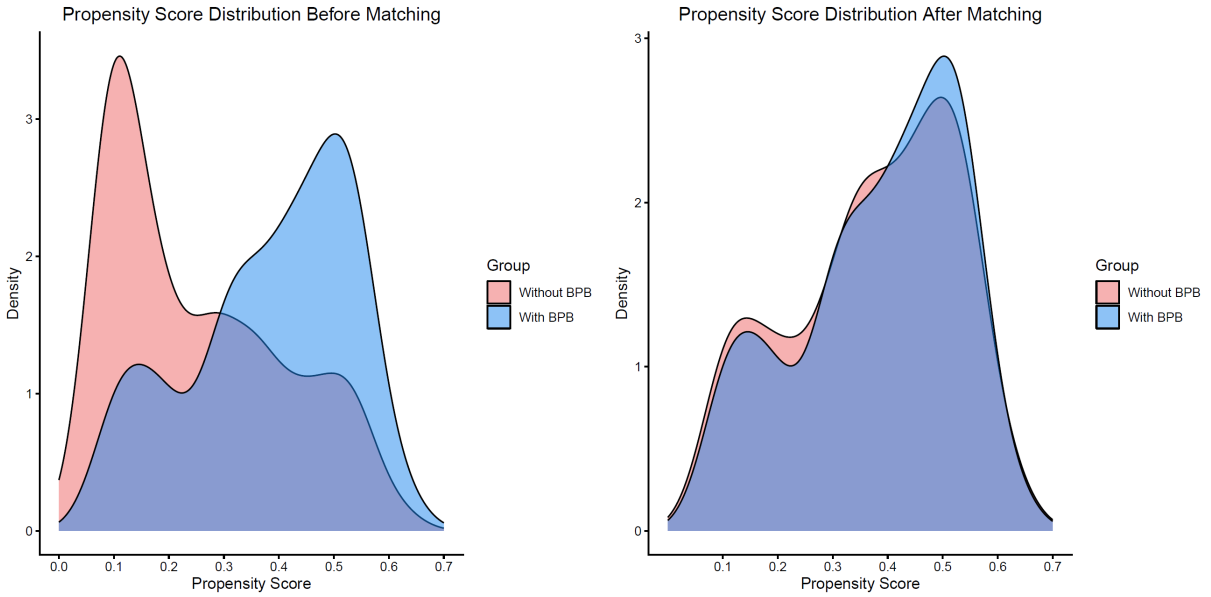

Supplementary Figure S1. (A) Propensity score distribution in both groups before matching. (B) Propensity score distribution in both groups after matching. BPB: brachial plexus block.

Supplementary Table S1. Conditional logistic regression[1]

	With BPB	Without BPB	Risk Difference[3]	cOR (95% CI)[4]	*P* value	aOR (95% CI)[5]	*P* value
Intraoperative fentanyl use	33/205	89/205	-27.3%	0.27 (0.17-0.44)	<0.001	0.23 (0.13-0.40)	<0.001
Intraoperative ephedrine use	33/205	8/205	+12.2%	4.57 (2.02-10.4)	<0.001	7.45 (2.27-24.4)	<0.001
Intraoperative nicardipine use	15/205	29/205	-6.8%	0.48 (0.25-0.93)	0.030	0.46 (0.23-0.91)	0.025
Intraoperative labetalol use	7/205	27/205	-9.8%	0.17 (0.06-0.48)	<0.001	0.11 (0.02-0.50)	0.005
Intraoperative hypertension	118/205	158/205	-19.5%	0.39 (0.24-0.61)	<0.001	0.38 (0.23-0.63)	<0.001
Intraoperative hypotension	44/205	35/205	+4.4%	1.39 (0.81-2.38)	0.227	1.46 (0.80-2.68)	0.221
Rescue analgesics use at PACU[2]	21/205	109/205	-43.0%	0.08 (0.04-0.17)	<0.001	0.06 (0.03-0.16)	<0.001
Opioid analgesics use during hospitalization	76/205	161/205	-41.4%	0.16 (0.09-0.27)	<0.001	0.10 (0.05-0.21)	<0.001

[1]Odds ratios were calculated with patients who did not receive the brachial plexus block as the reference group.

[2]Rescue analgesics at PACU included intravenous morphine, fentanyl, tramadol, or ketorolac.

[3]Risk difference calculated as risk in with BPB group minus risk in without BPB group. Negative values indicate risk reduction associated with BPB.

[4]Univariable conditional logistic regression

[5]Multivariable conditional logistic regression adjusted for fracture site and anesthesia time whose standardized mean difference remained greater than 0.1 after propensity score matching.

aOR: adjusted odds ratio; BPB: brachial plexus block; CI: confidence interval; cOR: crude odds ratio; PACU: postanesthesia care unit

Supplementary Table S2. Univariable stratified cox regression[1]

	With BPB	Without BPB	Risk Difference[2]	cHR (95% CI)[3]	*P* value	aHR (95% CI)[4]	*P* value
Breakthrough morphine use in the ward	39/205	58/205	-9.2%	0.53 (0.34-0.83)	0.005	0.47 (0.29-0.78)	0.003

[1]Hazard ratio was calculated with patients receiving the brachial plexus block as the reference group.

[2]Risk difference calculated as risk in with BPB group minus risk in without BPB group. Negative values indicate risk reduction associated with BPB.

[3]Univariable conditional logistic regression

[4]Multivariable conditional logistic regression adjusted for fracture site and anesthesia time whose standardized mean difference remained greater than 0.1 after propensity score matching.

aOR: adjusted odds ratio; BPB: brachial plexus block; CI: confidence interval; CI: confidence interval; cOR: crude odds ratio; HR: hazard ratio

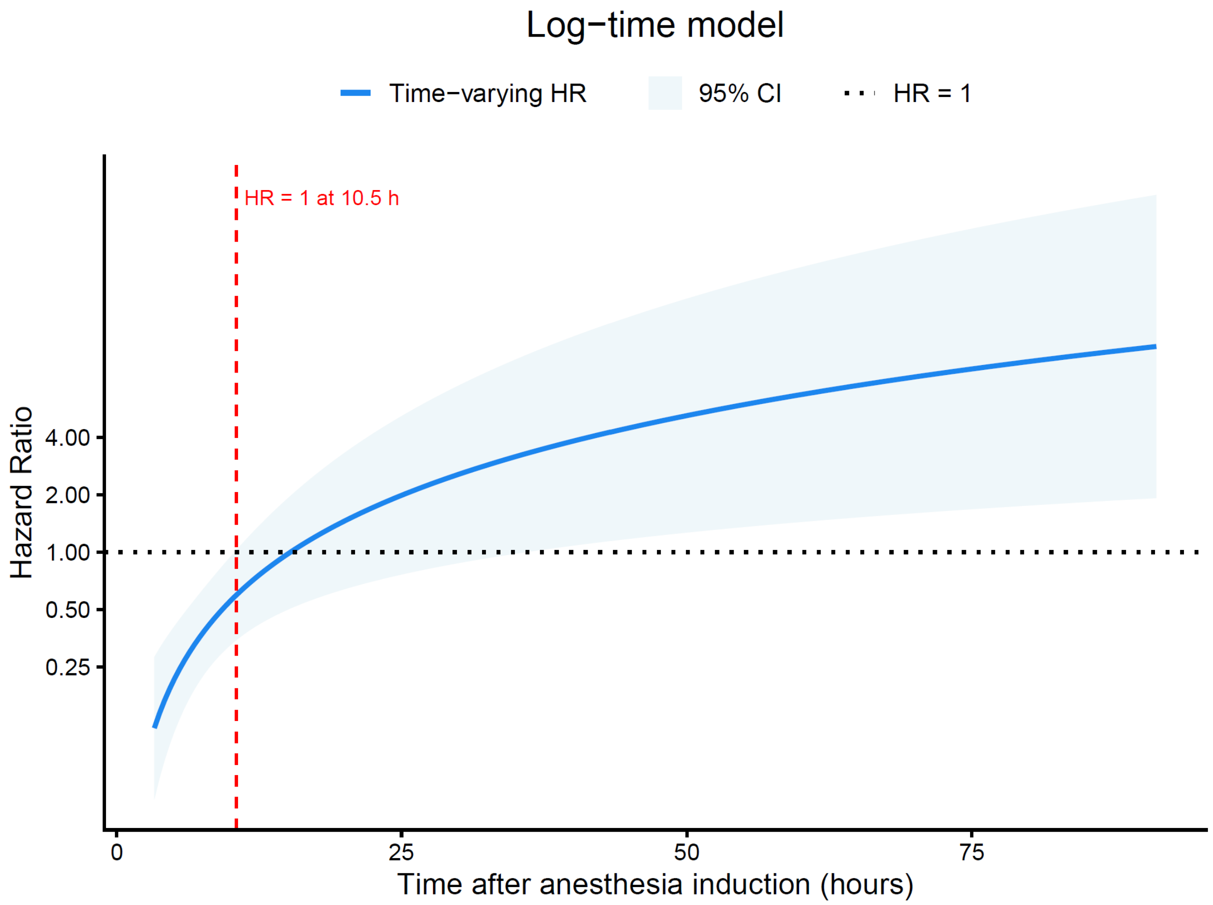

Supplementary Figure S2. **Time-varying hazard ratio with a log-time interaction model.** In the log-time interaction model, the interaction term was statistically significant (HR 4.61; 95% CI 2.21–9.62; P < 0.001), and the upper bound of the 95% CI crossed the null at approximately 10.5 hours after anesthesia induction. CI: confidence interval. HR: hazard ratio.

Supplementary Table S3. Area under curve of hemodynamic changes.

	Without BPB	With BPB	*P* value
Heart rate	504.5 ± 299.4	419.3 ± 320.8	0.006
Systolic blood pressure	1360.1 ± 890.8	972.7 ± 707.4	< 0.001
Diastolic blood pressure	789.3 ± 496.2	582.2 ± 387.1	< 0.001
Mean arterial pressure	979.3 ± 636.5	707.2 ± 512.5	< 0.001

Data are presented as mean ± standard deviation.

Supplementary Table S4. Association of brachial plexus block approaches, ropivacaine dose and volume on binary outcomes.

Outcome	Variable	aOR (95% CI)[1]	P value
Intraoperative fentanyl use	Interscalene	0.38 (0.06-2.18)	0.279
	Supraclavicular	0.60 (0.21-1.74)	0.332
	Ropivacaine dose (per 10 mg)	0.70 (0.49-0.98)	0.041
	Total injectate volume (per 5 mL)	0.82 (0.23-3.05)	0.757
Intraoperative ephedrine use	Interscalene	0.95 (0.06-25.40)	0.973
	Supraclavicular	0.27 (0.10-0.70)	0.008
	Ropivacaine dose (per 10 mg)	0.84 (0.58-1.19)	0.326
	Total injectate volume (per 5 mL)	13.00 (1.89-271)	0.027
Intraoperative nicardipine use	Interscalene	1.34 (0.11-19.60)	0.819
	Supraclavicular	1.20 (0.23-9.23)	0.836
	Ropivacaine dose (per 10 mg)	1.24 (0.78-2.02)	0.363
	Total injectate volume (per 5 mL)	0.34 (0.05-2.08)	0.240
Intraoperative labetalol use	Interscalene	6.33 (0.003-10340)	0.601
	Supraclavicular	8.25 (0.27-1210)	0.307
	Ropivacaine dose (per 10 mg)	0.66 (0.24-1.59)	0.368
	Total injectate volume (per 5 mL)	5.4 (0.20-410)	0.360
Intraoperative hypertension	Interscalene	4.71 (1.05-22.40)	0.046
	Supraclavicular	1.53 (0.70-3.36)	0.286
	Ropivacaine dose (per 10 mg)	0.99 (0.76-1.30)	0.957
	Total injectate volume (per 5 mL)	1.39 (0.46-4.18)	0.552
Intraoperative hypotension	Interscalene	0.13 (0.02-0.75)	0.027
	Supraclavicular	0.60 (0.23-1.54)	0.279
	Ropivacaine dose (per 10 mg)	0.86 (0.63-1.18)	0.343
	Total injectate volume (per 5 mL)	0.86 (0.26-2.86)	0.801
Rescue analgesics use at PACU	Interscalene	6.25 (0.67-77.80)	0.122
	Supraclavicular	4.00 (0.95-28.00)	0.09
	Ropivacaine dose (per 10 mg)	0.90 (0.60-1.35)	0.601
	Total injectate volume (per 5 mL)	1.55 (0.32-9.17)	0.602
Opioid analgesics use during hospitalization	Interscalene	1.06 (0.24-4.54)	0.942
	Supraclavicular	1.32 (0.61-2.96)	0.487
	Ropivacaine dose (per 10 mg)	0.76 (0.58-0.98)	0.038
	Total injectate volume (per 5 mL)	2.06 (0.70-6.41)	0.198

[1]Multivariable logistic regression adjusted for brachial plexus block approaches, ropivacaine dose, total injectate volume, parecoxib, paracetamol, emergency surgery status, year of operation, operative time, estimated blood loss, and fracture site. Brachial plexus block approaches were

compared with the axillary block as the reference group.
aOR: adjusted odds ratio; CI: confidence interval; PACU: post-anesthesia care unit.

Supplementary Table S5. Association of brachial plexus block approaches, ropivacaine dose and volume on continuous outcomes.

**Outcome**	**Variable**	**aMD (95% CI)**[1]	***P* value**
MME at PACU	Interscalene	0.75 (-0.33, 1.82)	0.171
	Supraclavicular	0.48 (-0.11, 1.06)	0.108
	Ropivacaine dose (per 10 mg)	-0.06 (-0.26, 0.13)	0.515
	Total injectate volume (per 5 mL)	0.34 (-0.45, 1.14)	0.395
Total MME	Interscalene	0.38 (-1.81, 2.57)	0.734
	Supraclavicular	0.72 (-0.47, 1.92)	0.233
	Ropivacaine dose (per 10 mg)	-0.22 (-0.61, 0.18)	0.285
	Total injectate volume (per 5 mL)	0.60 (-1.03, 2.23)	0.470
First VAS pain score in the ward	Interscalene	0.36 (-0.54, 1.26)	0.434
	Supraclavicular	0.02 (-0.47, 0.51)	0.938
	Ropivacaine dose (per 10 mg)	0.06 (-0.10, 0.22)	0.478
	Total injectate volume (per 5 mL)	0.32 (-0.36, 0.99)	0.355

[1]Multivariable logistic regression adjusted for brachial plexus block approaches, ropivacaine dose, total injectate volume, parecoxib, paracetamol, emergency surgery status, year of operation, operative time, estimated blood loss, and fracture site. Brachial plexus block approaches were compared with the axillary block as the reference group.

aMD: adjusted mean difference; CI: confidence interval; MME: morphine milligram equivalents; PACU: postanesthesia care unit; VAS: visual analogue scale.

Supplementary Table S6. Association of brachial plexus block approaches, ropivacaine dose and volume on time-to-event outcomes.

**Outcome**	**Variable**	**aHR (95% CI)[1]**	***P* value**
Breakthrough morphine use in the ward	Interscalene	3.61 (0.32-41.00)	0.301
	Supraclavicular	1.75 (0.80-3.82)	0.160
	Ropivacaine dose (per 10 mg)	0.96 (0.73-1.27)	0.783
	Total injectate volume (per 5 mL)	5.26 (0.64-43.10)	0.122

[1]Multivariable logistic regression adjusted for brachial plexus block approaches, ropivacaine dose, total injectate volume, parecoxib, paracetamol, emergency surgery status, year of operation, operative time, estimated blood loss, and fracture site. Brachial plexus block approaches were compared with the axillary block as the reference group.
aHR: adjusted hazard ratio; CI: confidence interval.

Supplementary Table S7. Sensitivity analysis assessing the potential impact of brachial plexus block failure on the primary outcomes.

Outcomes	5% Failure Rate			10% Failure rate			15% Failure Rate		
	Median estimate	Empirical 95% Interval	Significance Rate	Median estimate	Empirical 95% Interval	Significance Rate	Median estimate	Empirical 95% Interval	Significance Rate
Intraoperative fentanyl use	0.27	0.20, 0.35	100.00%	0.37	0.20, 0.37	100.00%	0.29	0.19, 0.41	100.00%
Intraoperative ephedrine use	4.00	2.50, 8.00	100.00%	7.50	2.23, 7.50	99.40%	3.37	1.96, 9.33	97.00%
Intraoperative nicardipine use	0.48	0.35, 0.67	73.00%	0.69	0.32, 0.69	56.80%	0.50	0.30, 0.72	48.40%
Intraoperative labetalol use	0.22	0.13, 0.31	100.00%	0.37	0.13, 0.37	99.60%	0.25	0.12, 0.39	97.80%
Intraoperative hypertension	0.40	0.30, 0.52	100.00%	0.56	0.30, 0.56	99.80%	0.43	0.30, 0.58	99.40%
Intraoperative hypotension	1.24	0.97, 1.64	1.40%	1.62	0.93, 1.62	1.20%	1.22	0.86, 1.77	2.40%
Rescue analgesics use at PACU	0.12	0.06, 0.16	100.00%	0.18	0.06, 0.18	100.00%	0.12	0.07, 0.18	100.00%
Opioid analgesics use during hospitalization	0.19	0.12, 0.26	100.00%	0.28	0.13, 0.28	100.00%	0.21	0.13, 0.30	100.00%
Breakthrough morphine use in the ward	0.57	0.44, 0.73	80.00%	0.77	0.42, 0.77	72.40%	0.57	0.40, 0.78	67.80%
MME at PACU	-2.05	-2.33, -1.78	100.00%	-1.69	-2.32, -1.69	100.00%	-1.98	-2.34, -1.63	100.00%
Total MME	-4.13	-4.62, -3.61	100.00%	-3.50	-4.69, -3.50	100.00%	-3.97	-4.62, -3.28	100.00%
First VAS pain score in the ward	-0.93	-1.1, -0.79	100.00%	-0.72	-1.12, -0.72	100.00%	-0.88	-1.09, -0.66	100.00%

MME: morphine milligram equivalents; PACU: postanesthesia care unit; VAS: visual analogue scale
